# Levodopa–Carbidopa–Entacapone Intestinal Gel for Advanced Parkinson’s Disease—Results from a Monocentric Study Evaluating Both Motor and Non-Motor Manifestations

**DOI:** 10.3390/biomedicines13092191

**Published:** 2025-09-08

**Authors:** Mihaiela Lungu, Violeta Diana Oprea, Luminița Lăcrămioara Apostol, Eva Maria Elkan, Ana Maria Ionescu, Anca Tudor, Lucian Andrei Zaharia

**Affiliations:** 1 Multimodal Parkinson’s Disease Treatment Center, Neurological Department, “St. Ap. Andrew” County Emergency Clinical Hospital in Galații, 800578 Galati, Romania; micalungu@gmail.com; 2 Clinical Medical Department, Faculty of Medicine and Pharmacy, “Dunarea de Jos” University of Galati, 800008 Galati, Romania; 3 Geriatric Department, Sjællands Universitetshospital Nykøbing Falster, 4800 Nykøbing Falster, Denmark; 4 Medical Department, Faculty of Medicine and Pharmacy, “Dunărea de Jos” University of Galati, 800008 Galati, Romania; 5 “St. Ap. Andrew” County Emergency Clinical Hospital in Galați, 800578 Galati, Romania; lum.apostol@gmail.com; 6 Department of Neuropsychiatry, “St. John” Clinical Emergency Hospital for Children, 800487 Galati, Romania; cojocarumariaeva@yahoo.com; 7 Morphological and Functional Sciences Department, Faculty of Medicine and Pharmacy, “Dunarea de Jos” University of Galati, 800008 Galati, Romania; 8 Faculty of Medicine, University Ovidius Constanța, 900470 Constanța, Romania; iuliusana@gmail.com; 9 Medical Informatics and Biostatistics, Research Center in Dental Medicine Using Conventional and Alternative Technologies, Faculty of Dental Medicine, “Victor Babes” University of Medicine and Pharmacy, 300041 Timisoara, Romania; atudor@umft.ro; 10 Neurological Department, “St. Ap. Andrew” County Emergency Clinical Hospital in Galați, 800578 Galati, Romania

**Keywords:** advanced Parkinson’s disease, levodopa–carbidopa–entacapone intestinal gel, device-assisted therapy

## Abstract

**Background**: Parkinson’s disease (PD) in advanced stages becomes, over time, a significant challenge, as oral medication becomes ineffective, and it may become necessary to switch to device-assisted therapy (DAT). This should be personalized according to the stage of the disease, the cognitive status of the patients, the association of frailty syndrome or other comorbidities, the support in care from the family, etc. Levodopa–carbidopa–entacapone intestinal gel can significantly improve the status of patients, provided that they are correctly selected for this type of treatment. **Materials and Methods**: We conducted a single-center prospective study including 20 advanced PD patients, who received a levodopa–carbidopa–entacapone gel through an intestinal pump, within the Parkinson’s Disease Multimodal Treatment Center of the Neurology Clinic of the “St. Ap. Andrew” County Emergency Clinical Hospital in Galați, Romania. The evaluations were performed at baseline (T0), after intestinal pump insertion (T1), and 6 months after the procedure (T2). **Results**: In the study group, the administration of the levodopa–carbidopa–entacapone intestinal gel, using the device for intestinal administration, had significant benefits, especially for motor symptoms. The periods of off, no-on, freezing, sudden-off, as well as dyskinesia and morning akinesia, were significantly reduced. Among the non-motor symptoms, depression and sleep disorders improved, with no changes in cognitive status and psychotic disorders. **Conclusions**: Adding new data for the use of device-assisted therapy in advanced PD, our study also highlights the need to further research this challenging patient profile.

## 1. Introduction

Although Parkinson’s disease (PD) causes major disability, our knowledge is still lacking in terms of fully understanding the disease, including complete pathways in the development and progression of the disease, as well as the heterogeneous clinical manifestations and sometimes unpredictable disease course. Multiple pathophysiological hypotheses fail to establish what exactly triggers the disease and, as a result, we still do not have any effective cure. PD is one of the fastest growing neurological disorders globally [1,2,3]. Dopaminergic neuron degeneration in the substantia nigra pars compacta is the hallmark of PD pathophysiology, while some of the major hypotheses for the underlying causes are multifactorial and still not fully understood [3,4,5].

Parkinson’s disease manifestations include primary motor symptoms (tremor, bradykinesia, muscle rigidity, poor balance) and also non-motor features. Typically, symptoms start gradually and worsen over time, affecting movement, mental health, sleep, and other bodily functions [6,7].

In the absence of an etiological treatment, the gold-standard symptomatic treatment remains levodopa. Its efficacy is enhanced by the addition of dopaminergic agonists, MAO inhibitors, and COMT inhibitors, but even so, they may become inadequate for oral administration over time.

Oral levodopa can become less effective over time or show reduced efficacy in certain patients for various reasons: disease progression triggering loss of dopaminergic neurons and the narrowing of the therapeutic window, gastrointestinal issues impairing drug absorption (gastroparesis, small intestinal bacterial overgrowth (SIBO)) but also its bioavailability (for example, competition with dietary proteins), pharmacokinetic variability, insufficiency of DOPA decarboxylase inhibitors, levodopa-induced complications (motor fluctuations, dyskinesias), non-motor symptoms (like cognitive impairment or depression that influence the overall perception of efficacy), drug–drug interactions, or poor adherence to therapy or dosing errors. Gastroparesis slows levodopa absorption through delayed gastric emptying [3,5,7].

Orally administered medication can become ineffective. Patients will experience reduced duration of *on*, extended *off*, *no-on*, or freezing, along with dyskinesia and painful dystonia, and worsening of non-motor symptoms. 

In patients with significant motor fluctuations and dyskinesia, device-assisted therapy (DAT) can offer efficient therapeutic alternatives, such as deep brain stimulation (DBS), a subcutaneously administered apomorphine pump—currently considered equivalent to levodopa—or an intrajejunal infusion pump for the gel containing levodopa–carbidopa–entacapone. The three components of the gel are delivered and absorbed at the jejunal level, and the carbidopa and entacapone components augment the effect of levodopa. While intestinal pumps can be suppressed at any time, DBS has the disadvantage of permanent intracerebral electrodes [6,8,9,10,11].

Patients with PD may be referred for DAT when they experience significant motor fluctuations and dyskinesias that interfere with their quality of life, despite maximal oral or transdermal levodopa treatment. Specific inclusion criteria for DAT refer to patients who require levodopa more than 4–5 times daily, experience troublesome “off” periods for more than 2 h a day, or have significant dyskinesias for more than 1 h daily [1,3,6]. Recently, the combination of foslevodopa/foscarbidopa continuous subcutaneous infusion (CSCI) was introduced in some countries as a treatment for advanced Parkinson’s disease (APD) patients experiencing motor fluctuations. CSCI is a soluble formulation of levodopa and carbidopa prodrugs, administered via a pump, and designed to provide 24 h symptom control [6,7,12,13,14,15]. The PD intestinal gel containing levodopa–carbidopa–entacapone combination therapy is licensed for the treatment of advanced PD with severe motor fluctuations and hyperkinesia or dyskinesia when available oral combinations of Parkinson medicinal products have not given satisfactory results [14,15,16,17].

## 2. Materials and Methods

Within the Multimodal Parkinson’s Disease Treatment Center affiliated with the Neurology Clinic of the “St. Ap. Andrew” County Emergency Clinical Hospital in Galați, Romania, we conducted a prospective, single-center study including 20 consecutive patients with advanced PD, previously on oral PD medication that became ineffective at the maximal dose. The narrowing of the therapeutic window and gastroparesis as an important non-motor vegetative manifestation of the disease, together with other issues related to APD, caused these patients to become non-responsive to their oral medication. Exclusion criteria: patients with dementia, lack of informed consent requirements (for example, patients without relatives/legal guardians), current/medical history of neoplastic diseases. The study was conducted for 12 months between April 2023 and April 2024.

All patients were carefully screened, starting from the 5-2-1 status (5 doses of levodopa daily, with 2 h *off* and 1 h of dyskinesia)—the main inclusion criteria to define oral therapy inefficacy in patients presenting APD.

Demographic data, the period since the onset of the disease, the stage of the disease on the Hoehn and Yahr (Ho–Ya) scale, and screening for the cognitive and depressive status were analyzed, and supplementary assessments and tests were performed. The database analysis for the 20 patients included histopathological background, chronic medication, the time of diagnosis and evolution of PD, and information about the oral medication at the time of enrollment. Patients were examined at 3 relevant milestones: T0—at study enrollment; T1—immediately after installing the device; and T2—6 months after the intervention.

Comorbidities, both at baseline and during the study, were also carefully noted and adequately treated as per local medical standards and protocols.

Standard validated tools were used: the Hoehn and Yahr scale, MADRS, and MMSE scale. The MDS-UPDRS I-IV scales are not standard in the local protocols for PD assessment, so they could not be used as study design parameters. All patients were evaluated by cerebral computed tomography (CTC); blood tests—tumor markers (CEA, PSA, CA 19-9), thyroid function (TSH, freeT4, freeT3), blood count, liver and kidney function tests, blood glucose—were also performed. Also, vitamin B12 and vitamin D plasma levels were measured.

Data were collected in the *off* medication state. We tracked the duration of the periods of *on* and *off*, the state of *no-on*, the number and duration of freezing events, and the type and duration of dyskinesia and dystonia before and after fitting the levodopa–carbidopa–entacapone therapy gel pump.

The required dose of levodopa was calculated, starting from the therapeutic oral medication of each patient and evaluating the necessary equivalences [17]. After the patients’ inclusion in the study group, the therapeutic test was performed by inserting, under sedation—analgesia—a nasojejunal (NJ) tube, to which the levodopa–carbidopa–entacapone intestinal gel therapy pump was attached.

For each patient the morning dose was titrated, as well as the continuous dose and the extra doses. The levodopa-equivalent dose (LED) was identified as the amount of levodopa (associated with carbidopa) that had a similar effect as the drug taken. Adding together all the LEDs in a day gives the levodopa-equivalent daily dose (LEDD) in every patient.

If the response to the therapeutic test was favorable, a gastrojejunostomy, with the insertion of the percutaneous endoscopic gastrostomy–jejunostomy tube (PEG-J), was performed, with the permanent fitting of the device, which established effective therapeutic doses.

We registered the occurrence and duration of the phenomena of *on*, *no-on*, *off*, freezing, and dyskinesia and dystonia, but also of the non-motor symptoms: depression, anxiety, sleep disorders, sensitive neuropathy, any psychotic elements, and hallucinations. All motor symptoms were evaluated in a specialty neurology department by clinical observation and posture assessment, together with standardized tools (like, for example, HY scale, Tinetti test, gait assessment tools, etc.).

Following the criteria of frailty syndrome, i.e., Fried’s Frailty Phenotype, and also assessing the Frailty Index (which includes age over 75 years, altered mobility and nutrition with frequent falls and secondary post-traumatic pathology, cognitive dysfunction, anxiety and depressive disorders, sarcopenia, vitamin D and other oligoelements deficiencies, social isolation, comorbidities/chronic diseases), we found that all the patients included in the study also presented, in addition to the diagnosis of PD, the criteria for frail elderly.

Statistical analysis was based on the IBM SPSS software v.29 (2022), using the following methods: test of normality, Shapiro–Wilk test, Friedman nonparametric test, Wilcoxon signed-rank test.

The study was conducted in accordance with the Declaration of Helsinki and approved by the Ethics Committee of the “St. Ap. Andrew” County Emergency Clinical Hospital in Galați, Romania (6954/8 April 2025), in accordance with local and international laws and regulations.

There was no use of AI at any point in the data analysis or article development.

## 3. Results

### 3.1. Patient Group Description

Patient group demographics: 45% women and 55% men, with the mean age of female patients 62.66 years (53–72 years), and men 71.09 years (54–74). The average time interval from the onset of the disease to the initiation of levodopa–carbidopa–entacapone therapy gel pump was 11 years. 

Before the initiation of gel therapy, patients enrolled in the study received oral therapies with dopaminergic agonists (95%), MAO-B inhibitors (60%), COMT inhibitors (10%), amantadine (20%), levodopa–carbidopa–entacapone (25%), and levodopa–carbidopa (30%).

**Figure 1:**
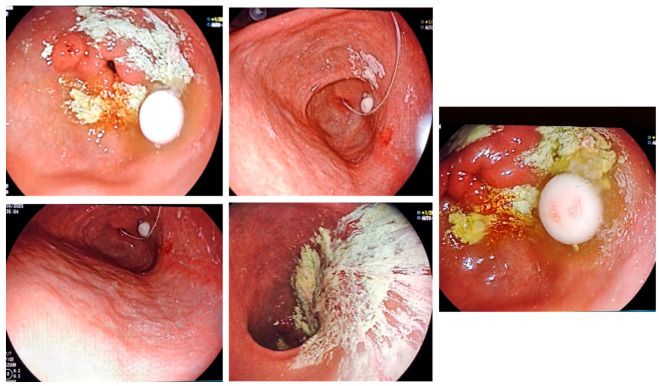
Gastroparesis in PD is preventing gastric transit for oral medication and trigger lack of efficacy—endoscopic images.

All patients presented with APD, some with gastroparesis-related oral therapy lack of efficacy (Figure 1).

### 3.2. Motor Symptom Results

The reduction in the score on the Hoehn and Yahr scale was significant after the administration of the levodopa–carbidopa–entacapone therapy gel, corresponding to a statistically significant efficacy in reducing the relevant PD symptoms. Thus, the values at the initiation of treatment and after 6 months show a decrease in the severity of Parkinson’s symptoms (Table 1).

While the levodopa doses varied from patient to patient, overall the doses were high, close to the maximum allowed. The median amount of levodopa-equivalent daily dose (LEDD) in the study was initially LEDD 0 = 1380.50, while 6 months after gel pump insertion (T2) we registered LEDD 1 = 1357.45.

We can conclude that the patient population required higher doses of levodopa–carbidopa–entacapone therapy gel for rebalancing. Thus, in our study LEDD 1 was not significantly reduced, but generally the treatment allowed the doses of antidepressant or antipsychotic drugs to be reduced (Table 2).

Periods of *on* initially had a median of 4.90 h, while 6 months after levodopa–carbidopa–entacapone gel therapy this significantly increased: 12.60 h (Table 3).

The *off* medication state initially measured 9.65 h on average, and 6 months after administration of the levodopa–carbidopa–entacapone therapy gel (T2), this was statistically significantly reduced to 0.785 h.

The phenomena *no-on*, that was initially presented by 60% of our patients, after the administration of the levodopa–carbidopa–entacapone jejunal infusion therapy, registered a significantly reduced duration. Furthermore, only 10% of patients maintained *no-on* phenomena at T2.

As for *freezing* episodes, we found a significant decrease in their frequency, duration, and intensity, from an initial average of 4.40 h at T0 to 0.75 h at T2 (6 months after the initiation of intestinal gel therapy), with some patients being free from the *freezing* state. The *freezing* phenomena initially manifested in our patients during stressful situations (especially when passing through narrow spaces), and five of the patients (20%) experienced repeated falls, with secondary trauma (one patient with vertebral and rib fractures).

Peak-dose choreiform-type dyskinesia was noted initially in eight patients (40%), with secondary gait impairment. At T2, under continuous administration and after dose adjustment, dyskinesia was present only in two patients (10% of the total study group) and with a shorter duration.

In our study group, dystonia was present in five patients (25%): painful dystonia of the leg—three patients (15%); oropharyngeal dystonia with dysphagia and phonation disorder—one patient (5%); and also one patient (5%) presented facial and latero-cervical dystonia, with grimaces and contractures in the sternocleidomastoid and trapezius muscles. After administration of levodopa–carbidopa–entacapone intestinal gel, only the patient with facial and later cervical dystonia maintained the symptoms, uninfluenced by the treatment. But, due to the small number of patients with dystonia in our group, we did not reach the statistical significance for the results to be able to conclude the efficacy of dystonic manifestations.

Postural instability was initially presented by nine patients (45%), with repeated episodes of falls in two cases, which improved after levodopa–carbidopa–entacapone gel therapy at the 6-month evaluation; at T2 it was only noted in two patients (10%). Oligokinesia and postural tremor were present in all patients in the group at the initiation of treatment (T0). After 6 months of levodopa–carbidopa–entacapone therapy, this was reduced considerably. 

After the initiation of the levodopa–carbidopa–entacapone intestinal gel therapy, we noticed a significant improvement in motor symptoms, with a decrease in *off*, *no-on*, freezing, *sudden-off*, and falls.

Management using the levodopa–carbidopa–entacapone intestinal gel significantly improved morning akinesia, freezing, and brady-hypo-oligokinesia. Dyskinesia was registered in some cases at the initiation of treatment (T1), before the therapeutic dose was optimized.

### 3.3. Vitamin Supplementation

We noted a vitamin D deficiency in 95% of the patients at the initial T0 baseline, with this type of deficiency being found in many degenerative diseases, mostly associated with the frailty syndrome in the elderly population.

Regarding vitamin B12 deficiency, at T0 we found low levels in two patients that did not receive continuous supplementation beforehand (10%). They presented clinical sensitive neuropathy type alterations, without electromyographic confirmation. All these patients received B vitamin supplementation and neurotrophic factors (cerebroprotein hydrolysate concentrate infusions administered intravenously; 10 mL during 10 days) during the study. 

### 3.4. Adverse Events

In our group, patients kept their intestinal pump active for an average of 16 h/day, and they received a dose of extended-release levodopa for the night.

The insertion of the levodopa–carbidopa–entacapone intestinal gel pump therapy allowed a decrease in doses of dopaminergic agonists.

The surgical periprocedural complications in our study group were as follows:

- Pneumoperitoneum—occurred in 95% of cases, with mild forms that did not required treatment.
- Extensive venous thrombosis in the mesenteric veins and deep venous system of a lower limb occurred in a patient, requiring administration of heparin by injectomate under APTT monitoring, with later oral anticoagulation regimen. The evolution was favorable, but the patient will remain permanently on new oral anticoagulant (NOAC) treatment.

As a result of this incident, we introduced into the local PEG-J insertion protocol the post-procedural administration of low-molecular-weight heparin, for a minimum of three days, with careful supervision of coagulation parameters.

We registered two drop-out cases. The first was a patient that died before the T2 6-month evaluation, due to an aggressive pancreatic adenocarcinoma—metastasis and death within one month after the oncologic diagnosis that occurred between the T1 and T2 timepoints of the study.

**Figure 2:**
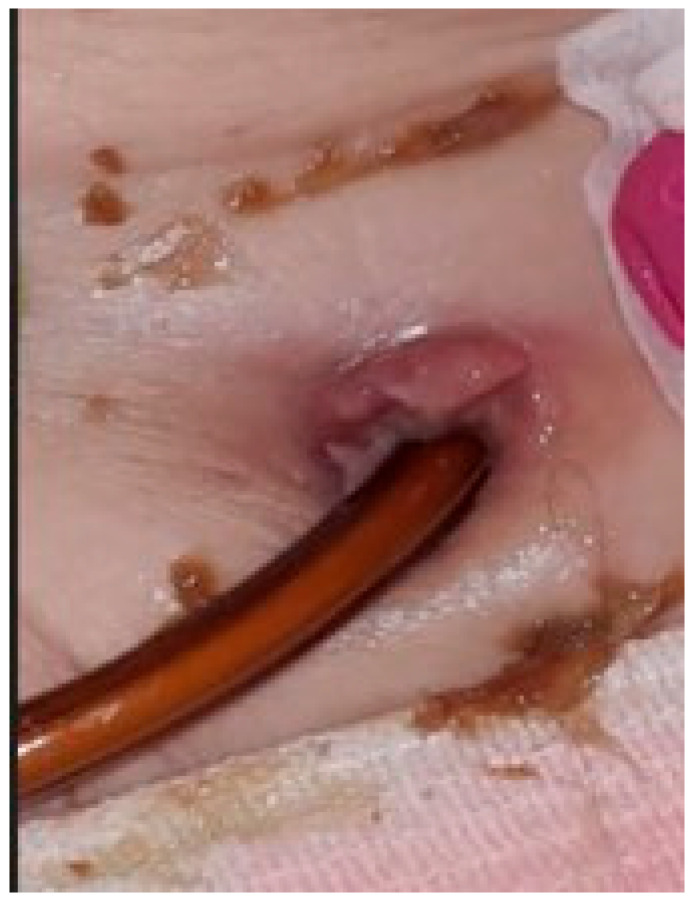
Infection at the level of gastrojejunostomy, triggered by poor hygiene, resulting in sepsis.

The second situation was a case of sepsis, with a starting point at the level of the stoma, in the context of diabetes mellitus and chronic kidney disease. The patient became estranged from her family shortly after surgery, due to an unforeseen situation; unfortunately she was unable to manage the device and the hygiene of her stoma herself (Figure 2). Therefore, the jejunal pump was suspended. Standard treatment was administered as per local protocols.

### 3.5. Non-Motor Symptoms

After the initiation of the levodopa–carbidopa–entacapone therapy gel, there was a significant improvement in both motor and non-motor symptoms, with a decrease in *off*, *no-on*, and freezing periods, but also with a decrease in dyskinesia, depressive state, and sleep disorders. The doses of antidepressants and antipsychotics were reduced in most cases.

The Mini-Mental State Examination (MMSE) and Montgomery–Asberg Depression Rating Scale (MADRS) were used for the assessment of psychiatric manifestations, known as clinical features, in PD. In our study group, two patients (10% of the total) presented at T0 with delirium of jealousy and guilt, which was significantly reduced after the levodopa–carbidopa–entacapone therapy pump was inserted, while the quetiapine treatment continued. The other 12 patients (60%) presented anxiety–depressive disorders at T0, for which they were already undergoing treatment with serotonin reuptake inhibitors. For them, too, the symptoms improved under levodopa–carbidopa–entacapone therapy, while in both situations, the doses of antipsychotics and antidepressants were significantly reduced before the follow-up evaluation (T2). Based on patients’ and families’ subjective evaluations, sleep disorders also improved (90% of cases).

The favorable evolution was confirmed by the improvement of the score on the MADRS scale—mean MADRS at T0 = 17.45 and at T2 = 8.75.

The MMSE changes were not significant (as no PD dementia patients were included in the study group): mean MMSE at T0 = 26.65 and at T2 = 26.95.

The values of the LEDD variable followed a normal distribution (Shapiro–Wilk test, *p* > 0.05); the rest of the variables did not (the significance test applied for LEDD was parametric; Table 4).

### 3.6. Time Related Evolution—Nonparametric Friedman+ Wilcoxon Signed-Rank Test

Significant differences were noted between the Ho–Ya scale assessment values at the three timepoints (Friedman test, *p* < 0.001) Table 5.

The Ho–Ya values decreased significantly at the T1 and T2 timepoints (Wilcoxon signed-rank test, *p* < 0.001) 

The *off* values decreased significantly after pump insertion (T1) and at 6 months from baseline (T2) (Wilcoxon signed-rank test, *p* < 0.001); and at 6 months from intestinal levodopa-entacapone-carbidopa therapy initiation (T2) (Wilcoxon signed-rank test, *p* = 0.010).

Significant differences were noted between the *sudden off* values at the three timepoints (Friedman test, *p* < 0.001; Table 6a,b).

Akinesia values were significantly lower at T2 (Wilcoxon signed-rank test, *p* = 0.002) 6 months from baseline. Also, freezing symptoms and MADRS scores decreased significantly at T2 at 6 months from baseline (Wilcoxon signed-rank test, *p* < 0.001; Table 7a,b).

*Falls*

The proportion of falls was significantly higher at the initial time compared to after the pump was installed (Chi2—Fisher’s exact test, *p* = 0.031). 

*Sleep disorders*

The proportion of sleep disturbances was significantly higher at baseline T0 as compared to T1, after pump insertion (Chi2—Fisher’s exact test, *p* < 0.001).

There was one event of discontinuation of therapy due to treatment-related side effects: septic complications in one case, as presented above, related exclusively to failure to follow stoma care. The safety profile observed was in line with data from other published clinical studies regarding treatment with the levodopa–carbidopa–entacapone intestinal gel infusion.

While generally safe, PEG-J placement and use can be associated with complications, such as infections, tube dislocation or migration, and peristomal leakage—based on information already available from clinical experience. In our study, after the insertion, the dimensions and use of the levodopa–carbidopa–entacapone therapy gel pump were considered acceptable by all our patients, but some of our subjects were initially reluctant with regard to having the gastrojejunostomy. Informed decisions following thorough patient education and a personalized approach was the key to ensuring later compliance for optimal therapy results.

There was a significant subjective improvement in the quality of life, reported by both patients and their families: patients confirmed that they partially or completely regained their autonomy, even being able to perform some tasks in the household, to manage their finances, to reconnect with their community, or even to travel.

## 4. Discussion

Although PD was first described in the 19th century, disease-modifying therapies are still unavailable. As the duration of living with PD increases, the signs and symptoms evolve clinically as well as pathologically, determining a correlated increase in morbidity. APD is today pathologically defined in terms of progressive nigrostriatal dopaminergic neuronal loss, although we have evidence that the neuropathologic processes are not limited to the dopaminergic system [7,8,9,10,11,12,13,14]. In 2003, Braak et al. characterized progressive stages of the disease in a progressive, anatomical manner as pathologic aggregation of alpha-synuclein (aSyn) [15]. However, the clinical utility of this staging system has been questioned and most clinical trials are based almost exclusively on clinical markers of disease progression and/or simply disease duration since diagnosis; but PD is clinically a very heterogeneous disease, which limits this method of classification [14,16].

Advancing stages of the disease are also associated with increased complications, resulting in prolonged hospitalizations for those living with the disease, an increased caregiver burden, and a growing burden of healthcare costs [17,18,19,20,21]. However, there is a lack of homogeneity and clarity in standardized criteria of clinical progression, and it is unclear how clinical symptoms correlate with other biomarkers of advancing disease. To effectively develop disease-modifying therapies and identify areas of need and intervention, it is not only necessary to develop clearer means of diagnosing the disease earlier, but also to define and understand its various stages [21,22].

The administration of the levodopa–carbidopa–entacapone intestinal gel therapy in patients with APD brings important positive effects in reducing motor fluctuations, dyskinesia and dystonia, and morning akinesia, with a statistically significant reduction in the periods of off, no-on, freezing, and sudden-off, but also with improved sleep, reduction of anxiety and depression, etc. [18,19,20,21,22,23].

In our group we had no reports of the side effects cited in other studies, like diarrhea, nausea, vomiting, dizziness, and headache [8,9,10]. Also, we did not record any treatment-related alterations in the biological samples or the electrocardiogram (no changes for the QT interval) or signs or symptoms that occurred beyond the pre-existing ones.

The average age of the patients in our group was 68.9 years, as compared with other studies—i.e., 71.2 years [8], 71.5 years [10].

The average duration of disease progression in the patient group was 21.1 years, as compared with 14.4 years in Santos Garcia et al.’s study [12], 14.1 years in Senek M et al. [9], and 15.5 years in Ötman et al.’s study [10,11]. We can conclude that our patients were enrolled after a longer period of disease progression—this is probably due to delayed access to the procedure and the medication.

On the Hoehn Yahr (Ho–Ya) scale, the average value at patient enrollment was 4.20, compared to the score quoted by Nyholm and Jost, which was 4 [13]. After insertion of the levodopa–carbidopa–entacapone gel pump, these values were significantly reduced to an average of 2 immediately after the installation (T1), and 1.75 at 6 months after the initiation of intestinal treatment (T2).

In our group, the average dose of levodopa—LEDD 0—at the time of pump insertion (T1) was 1380.50, compared to the dose quoted by Öthman et al.—median: 1040 mg (range: 370–2000 mg/day). As the last dosage, these authors found a value of 1365 mg (range: 798–2478) [10,11]. Santos-Garcia et al. found that the necessary dose at the beginning of treatment was 1393.3 mg on average [12].

The 6-month (T2) LEDD was 1357.45 mg, with no significant decrease in dose. There were high doses of levodopa, explained by the narrowing of the therapeutic window and gastroparesis, which made the oral treatment ineffective. Other studies confirmed that after 6 months of levodopa–carbidopa–entacapone therapy, LEDD remains constant [10,11,12]. However, the therapy allowed us to reduce the doses of dopaminergic agonists and MAO-B inhibitors.

The subsequent dose of levodopa decreased, but without significant reductions, due to the initial advanced stage of the patients. The average value of LEDD 1 at 6 months after the installation of the pump (T2) was 1357.45 mg, with a median range similar to the necessary doses of gel intake in other studies [12,13].

The duration of the *on* period in the patients in our group was initially 4.90 h, with a statistically significant increase of 11.65 h after the administration of the therapy and to 12.60 h after pump fitting, which has also been confirmed in other studies [12,13,14,15,16].

The *off* medication state initially had an average value of 9.65 h, but after the initiation of intestinal gel, this was significantly reduced to 2.425 h at T1 and to 1.85 h at T2 after 6 months. Similar conclusions were drawn in the Elegance study as well as in some others [15,16,17,18].

The initial *sudden off* had an average value of 2.65 h; after installing the pump the value reached 0.80 h; and at 6 months it reached 0.785 h, a statistically significant decrease; data confirmed by other authors [8,9,10].

The initial duration of the *freezing* phenomenon had an average of 4.40 h; at T2 the value was 0.75 h, with a statistically significant decrease; confirmed by other studies [17,18,19,20,21,22,23].

The duration of *no-on* phenomena, initially seen in 12 patients (60% of the study group), is consistent with other trials’ results [14,16], as at baseline, T0, the *no-on* ratio was significantly higher (Chi2 test, *p* < 0.001) than after the initiation of levodopa–carbidopa–entacapone gel (T1), when the number of patients with *no-on* moments decreased to two (10% of the total); a statistically significant reduction. 

In the study group, we had five patients with dystonia at the T0 baseline (25% of the total), of which one patient with oropharyngeal dystonia had associated severe dysphagia and phonation disorders. After the administration of the intestinal gel, only a slight decrease in dystonic phenomena was registered (not statistically significant). In other studies, a significant reduction in dystonia has been reported [14,16]. APD is characterized by motor fluctuation (periods of poor mobility) and also different dyskinesias (peak dose, diphasic, dystonia) [7,20]. While not a direct treatment for dystonia, the reduction in motor fluctuations and dyskinesias provided with the intestinal gel therapy can significantly lessen the severity of dystonia symptoms. A possible explanation of the difference between our results regarding the decrease in dystonia versus other studies may lay in patient selection (longer PD progression period prior to the study) and a lack of an objective standardized measurement for dystonia (mainly patient assessment).

Vitamin D deficiency was identified at baseline in 95% of patients, so supplementation treatment was initiated, considering the potential involvement of vitamin D in the pathogeny of some degenerative diseases and for frailty syndrome in the elderly, in general. There are numerous studies that suggest a link between low levels of vitamin D and the risk of developing PD, as well as its progression [20,21,22]. Vitamin D is neuroprotective, helping to regulate inflammation and protect dopaminergic neurons in the substantia nigra, also influencing the expression of genes involved in the functioning of the central nervous system. Some researchers concluded, in a large populational analysis, that patients with PD usually have significantly lower levels of vitamin D compared to healthy people of a similar age [20,21,22]. Studies show that 26% of patients with PD are deficient in vitamin D, while 69% have insufficient plasma levels [20,21,22]. The short follow-up period and the small number of patients did not allow any significant conclusions regarding the effects of vitamin D corrections in our study group, but nevertheless the confirmation of vitamin D deficiency in the PD study group was considered relevant.

In the group we had only two patients with sensory neuropathy complaints (not confirmed by EMG), for whom neurotrophic factors and B vitamins were administered. For the patients in the study group, only two (10% of the total) presented plasma vitamin B12 values below the lower limit of normal. Hypovitaminosis B12 may be involved in the development of peripheral neuropathy; prolonged exposure to levodopa also increased plasma levels of malonic acid and homocysteine. The approach for this type of peripheral involvement may consist of the supplementation of vitamin B12, along with COMT inhibitors, which prevent the methionine cycle from using B vitamins to form homocysteine [3,23,24,25,26,27].

Regarding depressive status and sleep disorders, after the initiation of levodopa–carbidopa–entacapone gel therapy, a reduction in the severity of the symptomatology was noted, which allowed the doses of antidepressive medication to be reduced. Based on cognitive assessment, we concluded that MMSE scores were similar at all three evaluation points (T0, T1, and T2). The MADRS had an initial T0 mean value of 17.45 points and after the initiation of levodopa–carbidopa–entacapone therapy (T1) a mean value of 8.75, which supports the effect of a reduction in depressive symptoms after introducing a more efficient PD treatment. The proportion of depression was insignificantly higher at baseline (T0) compared to after treatment evaluation at T2 (Chi2—Fisher’s exact test, *p* = 0.113). In other studies aimed at analyzing the efficacy of levodopa–carbidopa–entacapone intestinal gel therapy, motor phenomena were mainly evaluated, rarely focusing on non-motor manifestations. While not the primary focus, some studies suggested that the continuous dopaminergic stimulation provided by the levodopa–carbidopa–entacapone therapy may have direct positive effects on patients’ mood, potentially reducing apathy and depressive symptoms [3,27,28,29]. The underlying pathophysiological mechanisms of PD depression are not clear—according to some research it may involve impaired cortico-striatal and limbic brain circuits, and also an imbalance between neurotransmitter systems (mainly serotoninergic, dopaminergic, and noradrenergic), neuroinflammation, and the dysregulation of neurotrophic factors [3,27,28,29,30].

The main limitations of our study come from the small number of patients, which may negatively impact the significance of some of the results, together with the relatively short period of follow-up of only 6 months after the PEG-J pump insertion. As a single-center trial, the data should be interpreted in the context of local clinical practice and protocols, as well as the work being in a multidisciplinary team of neurologists, gastroenterologists, and surgeons serving in the same hospital.

## 5. Conclusions

In our study, the introduction of levodopa–carbidopa–entacapone therapy significantly improved motor symptoms in APD: morning akinesia; freezing; brady-hypo-oligokinesia; and known phenomena such as *off*, *sudden-off*, *no-on*, and dyskinesia; further to this, some of the non-motor manifestations were also reduced after the initiation of the new regimen and medication device.

The levodopa–carbidopa–entacapone therapy, administrated as an intestinal gel, is an innovative combination treatment for patients with APD (especially in low-efficacy cases of oral therapy administration). The combination allows a more efficient absorption of levodopa and a reduction in doses, due to the presence of entacapone, which increases levodopa’s bioavailability. The benefits were observed on both motor and non-motor manifestations: episodes of *off*, freezing, and dyskinesia were significantly reduced and sleep and psychiatric symptoms were improved, as well as the quality of life of both the patients and their caregivers. The administration of levodopa–carbidopa–entacapone gel allowed reductions in dose of the dopaminergic agonist, MAO-B inhibitor, and also of antidepressive medication.

What we have learned from our experience regarding adverse-event management may prove valuable for other clinical settings: post-surgery stoma care is crucial; also post-surgery venous thrombosis may occur. In our study, the occurrence of post-procedural venous thrombosis triggered an update in our local therapy algorithm to include short-term small molecule heparin regimen after the pump insertion, associated with standard prophylactic antibiotic therapy. 

Further studies are needed to complete management recommendations in the longer term. Safety and quality of life need to be considered also, while most currently available studies are characterized by substantial heterogeneity of the sample sizes, the eligibility criteria, the duration, the type and frequency of other interventions, and the assessment tools for non-motor symptoms, as well as their severity and/or PD stage.

## Figures and Tables

**Table 1:** Hoehn and Yahr scale values.

Time of Assessment			Statistic	Std. Error
Ho–Ya initial (T0)	Mean		4.20	0.186
	95% Confidence Interval for Mean	Lower Bound	3.81	
		Upper Bound	4.59	
	5% Trimmed Mean		4.28	
	Median		4.00	
	Variance		0.695	
	Hours of Deviation		0.834	
	Minimum		2	
	Maximum		5	
	Range		3	
	Interquartile Range		1	
	Skewness		−1.018	0.512
	Kurtosis		1.080	0.992
Ho–Ya after inserting the PEG-J tube (T1)	Mean		2.00	0.162
	95% Confidence Interval for Mean	Lower Bound	1.66	
		Upper Bound	2.34	
	5% Trimmed Mean		2.00	
	Median		2.00	
	Variance		0.526	
	Hours of Deviation		0.725	
	Minimum		1	
	Maximum		3	
	Range		2	
	Interquartile Range		2	
	Skewness		0.000	0.512
	Kurtosis		−0.931	0.992
Ho–Ya 6 months after the surgical intervention (T2)	Mean		1.75	0.160
	95% Confidence Interval for Mean	Lower Bound	1.41	
		Upper Bound	2.09	
	5% Trimmed Mean		1.72	
	Median		2.00	
	Variance		0.513	
	Hours of Deviation		0.716	
	Minimum		1	
	Maximum		3	
	Range		2	
	Interquartile Range		1	
	Skewness		0.418	0.512
	Kurtosis		−0.826	0.992

**Table 2:** LEDD doses measured at T1 and T2 timepoints in the study.

	Descriptives		Statistics	Standard Error
LEDD T1 (total dose of levodopa at T1)	Mean		1380.50	103.630
	95% Confidence Interval for Mean	Lower Bound	1163.60	
		Upper Bound	1597.40	
	5% Trimmed Mean		1379.89	
	Median		1333.00	
	Variance		214,784.368	
	Hours of Deviation		463.448	
	Minimum		595	
	Maximum		2177	
	Range		1582	
	Interquartile Range		771	
	Skewness		0.217	0.512
	Kurtosis		−0.884	0.992
LEDD 1, 6 months after the surgical intervention (T2)	Mean		1357.45	77.192
	95% Confidence Interval for Mean	Lower Bound	1195.89	
		Upper Bound	1519.01	
	5% Trimmed Mean		1346.89	
	Median		1326.50	
	Variance		119,171.103	
	Hours of Deviation		345.212	
	Minimum		787	
	Maximum		2118	
	Range		1331	
	Interquartile Range		544	
	Skewness		0.594	0.512
	Kurtosis		−0.247	0.992

**Table 3:** *On* time values.

	Descriptives		Statistics	Standard Error
*On* at baseline (T0)	Mean		4.90	0.774
	95% Confidence Interval for Mean	Lower Bound	3.28	
		Upper Bound	6.52	
	5% Trimmed Mean		4.89	
	Median		5.00	
	Variance		11.989	
	Hours of Deviation		3.463	
	Minimum		0	
	Maximum		10	
	Range		10	
	Interquartile Range		6	
	Skewness		−0.092	0.512
	Kurtosis		−1.262	0.992
*On* at T1	Mean		11.65	0.704
	95% Confidence Interval for Mean	Lower Bound	10.18	
		Upper Bound	13.12	
	5% Trimmed Mean		11.72	
	Median		12.00	
	Variance		9.924	
	Hours of Deviation		3.150	
	Minimum		6	
	Maximum		16	
	Range		10	
	Interquartile Range		5	
	Skewness		−0.068	0.512
	Kurtosis		−1.091	0.992
*On* 6 months after the surgical intervention (T2)	Mean		12.60	0.755
	95% Confidence Interval for Mean	Lower Bound	11.02	
		Upper Bound	14.18	
	5% Trimmed Mean		12.78	
	Median		13.50	
	Variance		11.411	
	Hours of Deviation		3.378	
	Minimum		6	
	Maximum		16	
	Range		10	
	Interquartile Range		6	
	Skewness		−0.526	0.512
	Kurtosis		−1.151	0.992

**Table 4:** Tests of normality applied to evaluate both motor and non-motor manifestations.

	Kolmogorov–Smirnova ^a^			Shapiro–Wilk		
	Statistic	df	Itself.	Statistic	df	Itself.
Initial Ho–Ya (T0)	0.255	20	0.001	0.804	20	0.001
Ho–Ya after pump (T1)	0.250	20	0.002	0.815	20	0.001
Ho–Ya at 6 months (T2)	0.252	20	0.002	0.795	20	0.001
Initial MMSE (T0)	0.280	20	0.000	0.792	20	0.001
MMSE after 6 months (T2)	0.257	20	0.001	0.828	20	0.002
*On* initial (T0)	0.128	20	0.200 *	0.924	20	0.120
*On* after pump insertion (T1)	0.150	20	0.200 *	0.937	20	0.207
*On* at 6 months (T2)	0.211	20	0.020	0.860	20	0.008
*Off* initial assessment (T0)	0.126	20	0.200 *	0.944	20	0.291
*Off* after pump (T1)	0.267	20	0.001	0.767	20	0.000
*Off* at 6 months (T2)	0.258	20	0.001	0.671	20	0.000
*Sudden off* initial T0	0.223	20	0.010	0.871	20	0.012
*Sudden off* after the pump insertion (T1)	0.341	20	0.000	0.699	20	0.000
*Sudden off* at 6 months (T2)	0.340	20	0.000	0.705	20	0.000
*Freezing* initial assessment (T0)	0.141	20	0.200 *	0.944	20	0.290
*Freezing* after pump (T1)	0.369	20	0.000	0.719	20	0.000
LEDD 1 (T1)	0.089	20	0.200 *	0.966	20	0.675
LEDD 2 after 6 months (T2)	0.154	20	0.200 *	0.952	20	0.400
MADRS initial assessment (T0)	0.165	20	0.157	0.892	20	0.029
MADRS after pump (T1)	0.232	20	0.006	0.842	20	0.004

* This is a lower bound of true significance. ^a^ Lilliefors significance correction.

**Table 5:** Ho–Ya scale assessment.

	N	Mean	Standard Deviation	Minimum	Maximum	^a^ Mean Rank
Ho–Ya at T0	20	4.20	0.834	2	5	2.95
Ho–Ya at T1	20	2.00	0.725	1	3	1.65
Ho–Ya at T2	20	1.75	0.716	1	3	1.40

^a^ Friedman test.

**Table 6:** (a): Friedman test for the analysis of *sudden off* values: ranks. (b) Friedman test for the analysis of *sudden off* values: test statistics ^a^.

(a)				
		N	Mean Rank	Sum of Ranks
*Sudden off* T1—*Sudden off* T0	Negative Ranks	13 ^a^	7.85	102.00
	Positive Ranks	1 ^b^	3.00	3.00
	Ties	6 ^c^		
	Total	20		
*Sudden off* T2—*Sudden off* T0	Negative Ranks	13 ^d^	7.85	102.00
	Positive Ranks	1 ^e^	3.00	3.00
	Ties	6 ^f^		
	Total	20		
*Sudden off* T2—*Sudden off* T1	Negative Ranks	1 ^g^	1.00	1.00
	Ranks	0 ^h^	0.00	0.00
	Ties	19 ^i^		
	Total	20		
**(b)**				
		*Sudden off* T1—*Sudden off* T0	*Sudden off* T2—*Sudden off* T0	*Sudden off* T2—*Sudden off* T1
With		−3.155 ^b^	−3.141 ^b^	−1.000 ^b^
Asymp. Sig. (2-tailed)		0.002	0.002	0.317

^a^ Sudden OFF at T1 < Sudden OFF at T0; ^b^ Sudden OFF at T1 > Sudden OFF at T0; ^c^ Sudden OFF at T1 = Sudden OFF at T0; ^d^ Sudden OFF at T2 < Sudden OFF at T0; ^e^ Sudden OFF at T2 > Sudden OFF at T0; ^f^ Sudden OFF at T2 = Sudden OFF at T0; ^g^ Sudden OFF at T2 < Sudden OFF at T1; ^h^ Sudden OFF at T2 > Sudden OFF at T1; ^i^ Sudden OFF at T2 = Sudden OFF at T1.

**Table 7:** (a): Akinesia, freezing symptoms and MADRS scores at T2 (6 months from the start). (b) Akinesia, freezing symptoms and MADRS scores at 6 months from the start (T2): ranks.

(a)					
	N	Mean	Hours of Deviation	Minimum	Maximum
Initial Akinesia (T0)	20	0.95	0.605	0	2
Initial Freezing (T0)	20	4.40	2.909	0	10
Initial MADRS (T0)	20	17.45	13.189	1	43
Akinesia at T2	20	0.25	0.444	0	1
Freezing at T2	20	0.75	1.020	0	3
MADRS at T2	20	8.75	7.348	1	25
**(b)**					
			**N**	**Mean Rank**	**Sum of Ranks**
Akinesia at 6 months (T2)—Akinesia initially (T0)		Negative Ranks	11 ^a^	6.00	66.00
		Positive Ranks	0 ^b^	0.00	0.00
		Ties	9 ^c^		
		Total	20		
Freezing after pump insertion (T1)—Freezing initially (T0)		Negative Ranks	17 ^d^	9.00	153.00
		Positive Ranks	0 ^e^	0.00	0.00
		Ties	3 ^f^		
		Total	20		
MADRS after pump insertion (T1)—MADRS initially (T0)		Negative Ranks	17 ^g^	9.00	153.00
		Positive Ranks	0 ^h^	0.00	0.00
		Ties	3 ^i^		
		Total	20		

^a^ Akinesia at 6 months (T2) < initial (T0) akinesia. ^b^ Akinesia at 6 months (T2) > initial (T0) akinesia. ^c^ Akinesia at 6 months (T2) = initial (T0) akinesia. ^d^ Freezing after pump insertion (T1) < initial (T0) freezing. ^e^ Freezing after pump insertion (T1) > initial (T0) freezing. ^f^ Freezing after pump insertion (T1) = initial (T0) freezing. ^g^ MADRS after pump insertion (T1) < initial (T0) MADRS. ^h^ MADRS after pump insertion (T1) > initial (T0) MADRS. ^i^ MADRS after pump insertion (T1) = initial (T0) MADRS.

## Data Availability

The original contributions presented in this study are included in the article. Further inquiries can be directed to the corresponding author.

## Abbreviations

The following abbreviations are used in this manuscript:

PD	Parkinson’s disease
APD	Advanced Parkinson’s disease
DAT	Device-assisted therapy
UPDRS	Unified Parkinson’s Disease Rating Scale
IMAO	Monoamine oxidase inhibitors
COMT	Catechol-O-methyltransferase
Ho–Ya	Hoehn and Yahr scale
DBS	Deep brain stimulation
CTC	Cerebral computed tomography
NJ	Nasojejunal
PEG-J	Percutaneous endoscopic gastrostomy–jejunostomy
LEDD	Levodopa-equivalent daily dose
NOAC	New oral anticoagulant
MMSE	Mini-Mental State Examination
MADRS	Montgomery–Asberg Depression Rating Scale
PINK1	PTEN-induced kinase 1 (phosphatase and tensin homolog)
EMG	Electromyography
